# Supplementary material for: Physicochemical Changes of Heat-Treated Corn Grain Used in Ruminant Nutrition
Source: Animals (Basel). 2022 Aug 30;12(17):2234. doi: 10.3390/ani12172234 (PMC9454732; doi:10.3390/ani12172234)
Supplement: Supplementary file 1 [file animals-12-02234-s001.zip › animals-1876454-supplementary.pdf]

Table S1. Correlation between gelatinization, functional and pasting properties of unprocessed and heat treated corn.

	T_p	T_e	$\Delta T=T_e-T_o$	ΔH	DG	WAI	WSI	IV	PV	FV	T_g
T_o	0.999[a]	0.999[a]	0.938[b]	0.860	-0.860	-0.971[a]	-0.979[a]	-0.997[a]	0.869	0.772	0.999[a]
T_p		1.000[a]	0.950[b]	0.844	-0.843	-0.965[a]	-0.981[a]	-0.999[a]	0.857	0.775	0.998[a]
T_e			0.954[b]	0.837	-0.837	-0.962[a]	-0.984[a]	-1.000[a]	0.854	0.784	0.997[a]
$\Delta T=T_e-T_o$				0.655	-0.654	-0.855	-0.959[a]	-0.962[a]	0.718	0.809	0.934[b]
ΔH					-1.000[a]	-0.952[b]	-0.793	-0.826	0.966[a]	0.604	0.872
DG						0.951[b]	0.793	0.825	-0.967[a]	-0.604	-0.871
WAI							0.936[b]	0.957[b]	-0.955[b]	-0.747	-0.978[a]
WSI								0.985[a]	-0.851	-0.878	-0.982[a]
IV									-0.847	-0.787	-0.996[a]
PV										0.767	0.887[b]
FV											0.788

[a] Significant at $p\leq0.01$.

[b] Significant at $p\leq 0.05$.

onset temperature (T_o), peak temperature (T_p), endset temperature (T_e), and enthalpy change (ΔH) of starch gelatinization; ΔT – gelatinization temperature range; DG-degree of gelatinization; WAI-water absorption index; WSI-water solubility index; IV-initial viscosity, PV-peak viscosity, FV-final viscosity, T_g-gelatinization temperature
